# What Is the Fastest Way to Connect Stations to a Wi-Fi HaLow Network?

**DOI:** 10.3390/s18092744

**Published:** 2018-08-21

**Authors:** Dmitry Bankov, Evgeny Khorov, Andrey Lyakhov, Ekaterina Stepanova, Le Tian, Jeroen Famaey

**Affiliations:** 1 Institute for Information Transmission Problems, Russian Academy of Sciences, 127051 Moscow, Russia; bankov@iitp.ru (D.B.); lyakhov@iitp.ru (A.L.); stepanova@iitp.ru (E.S.); 2 Telecommunication Systems Lab, National Research University Higher School of Economics, 101000 Moscow, Russia; 3 IDLab, Department of Mathematics and Computer Science, University of Antwerp-Imec, 2020 Antwerp, Belgium; Le.tian@uantwerpen.be (L.T.); jeroen.famaey@uantwerpen.be (J.F.)

**Keywords:** Internet of Things, IEEE 802.11ah, Wi-Fi HaLow, authentication, link set-up

## Abstract

Wi-Fi HaLow is an adaptation of the widespread Wi-Fi technology for the Internet of Things scenarios. Such scenarios often involve numerous wireless stations connected to a shared channel, and contention for the channel significantly affects the performance in such networks. Wi-Fi HaLow contains numerous solutions aimed at handling the contention between stations, two of which, namely, the Centralized Authentication Control (CAC) and the Distributed Authentication Control (DAC), address the contention reduction during the link set-up process. The link set-up process is special because the access point knows nothing of the connecting stations and its means of control of these stations are very limited. While DAC is self-adaptive, CAC does require an algorithm to dynamically control its parameters. Being just a framework, the Wi-Fi HaLow standard neither specifies such an algorithm nor recommends which protocol, CAC or DAC, is more suitable in a given situation. In this paper, we solve both issues by developing a novel robust close-to-optimal algorithm for CAC and compare CAC and DAC in a vast set of experiments.

## 1. Introduction

The Internet of Things (IoT) is an important part of the future network infrastructure, providing connectivity to numerous autonomous devices, such as sensors and actuators. The multiplicity of such devices, the necessity to place them in remote places, and convenience considerations promote wireless connectivity for IoT scenarios. In turn, building a wireless network of sensors and actuators raises its own problems, such as energy efficiency, reliability, and timeliness of communications in case of a large number of devices connected to a common wireless (hence broadcast) channel. Thus, any wireless technology for IoT should have an appropriate design of low-layer protocols to consider these problems.

A good illustration of such considerations is the Wi-Fi HaLow technology [1], based on the IEEE 802.11ah standard, which has evolved as an attempt to meet the IoT requirements while keeping the ideology of Wi-Fi. It has introduced to Wi-Fi many new mechanisms that address the IoT peculiarities. For example, the Restricted Access Window (RAW) [2] can be used to group stations (STAs) and to assign them dedicated time intervals for transmission, thus decreasing contention for the channel and improving transmission reliability. Another good example is the Traffic Indication Map Segmentation [3] mechanism which can be used to improve the energy efficiency of Wi-Fi by grouping STAs and enabling them to receive only those network advertisements and packets which are related to their specific group while spending the rest of the time in the doze state.

These and many other novelties of Wi-Fi HaLow make it suitable for massive machine-type communications. However, before a Wi-Fi access point (AP) can use these novel features to control an STA, the STA needs to set up the link with it. During the link set-up, the AP learns of the STA’s existence, of its capabilities and its admissibility to the network, informs the STA of the network parameters and assigns it an identifier, namely Association ID (AID), used throughout the entire process of data exchange with this STA. Thus, the AP’s means to control an STA before the end of the link set-up are quite limited. Therefore, to transmit management frames needed for link set-up, the STAs have to use the basic channel access method, namely Carrier Sense Multiple Access with Collision Avoidance (CSMA/CA) with binary exponential backoff.

In traditional Wi-Fi networks, where the typical number of connecting STAs is small, such an approach is sufficient to provide fast link set-up. At the same time, in IoT networks, it is a feasible scenario when a big number of devices is trying to simultaneously set up the link with an AP, e.g., when an AP serving a group of STAs reboots after a malfunction or a blackout and all the STAs served by it have to reconnect. Another example requiring connection of multiple STAs at once is the scenario with mobile devices. For a high number of contending STAs, CSMA/CA suffers from collisions and provides poor performance. As the result, link set-up lasts for a long time and causes degradation of network performance for the already connected devices.

Fortunately, the IEEE 802.11ah standard describes two protocols aimed at reducing the contention during the link set-up process and making it faster. The first one is the Centralized Authentication Control (CAC) protocol, which allows the AP to periodically set the portion of STAs that may send requests for link set-up. The second one is the Distributed Authentication Control (DAC) protocol, which allows the STAs to spread their link set-up requests over random time intervals in a way similar to binary-exponential backoff. Both protocols have a list of parameters. Moreover, CAC needs to change them online. However, being a framework, the standard only describes the protocols as a set of primitives to provide fast link set-up, but it does not specify how to configure them in various scenarios.

Although several papers have already studied the problem of fast link set-up in IEEE 802.11ah networks, they have many drawbacks and limitations. This paper generalizes and significantly extends our previous work in this area [4,5,6]. An important contribution of the paper is a new algorithm to adaptively control parameters of CAC. In contrast to our previous work, the designed algorithm continues learning during the whole link set-up process, which makes it more robust in scenarios with changing contention conditions, e.g., when several groups of STAs start link set-up one-by-one. For DAC, we show that its performance slightly depends on most of its parameters. We also compare the performance of CAC and DAC in different scenarios.

The rest of the paper is organized as follows. Section 2 provides a brief description of channel access in Wi-Fi networks and main peculiarities of CAC and DAC. Section 3 analyses prior arts on link set-up in IEEE 802.11ah networks. In Section 4, we introduce our algorithm to control CAC parameters focusing on the differences with the previous version. In Section 5, we compare the performance of CAC and DAC in a vast set of scenarios. Specifically, we show that the performance of DAC is insensitive to many of its parameters. In addition, we demonstrate that the designed algorithm is much more efficient than DAC in different scenarios. Conclusions are given in Section 6.

## 2. Background on Link Set-Up in Wi-Fi HaLow Networks

Link set-up in IEEE 802.11ah networks is based on the same primitives as in ordinary IEEE 802.11 networks; therefore, in order to understand the peculiarities of link set-up in Wi-Fi HaLow, one should understand the basic association procedure in Wi-Fi networks. In this section, we briefly describe link set-up in infrastructure Wi-Fi networks, focusing on authentication and association procedures as well as on the default channel access method.

### 2.1. Link Set-Up in Wi-Fi Networks

**Figure 1:**
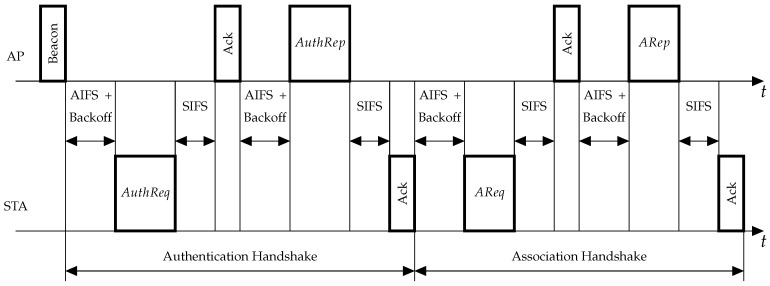
An example of link set-up handshakes.

Link set-up in infrastructure Wi-Fi networks consists of two major handshakes: authentication and association (see Figure 1). Authentication starts after the STA receives a beacon (periodically sent by the AP) or a Probe Response frame from the AP and thus learns of the AP and network presence. During the authentication handshake, the AP and the STA confirm their identity by exchanging security keys. For that, the STA sends an Authentication Request frame (AuthReq) to the AP, and the AP responds with an Authentication Response frame (AuthRep). Even if the network is not secure, i.e., the network uses Open System authentication, the authentication handshake is still mandatory.

The purpose of the association handshake is to let the STA inform the AP about its capabilities, e.g., supported modulation and coding schemes and channels, and to let the AP set the channel access parameters for the STA. In addition, during the association, the AP assigns an Association ID (AID) to the STA. AID is later used by the AP instead of the STA’s address as a short identifier in many control mechanisms. To associate with an AP, the STA sends an Association Request frame (AReq), to which the AP replies with an Association Response frame (ARep). Only after that may the STA transmit and receive data frames.

### 2.2. Channel Access

All frames involved in the authentication and association process, i.e., AuthReq, AuthRep, AReq and ARep, are transmitted using the default random channel access: Enhanced Distributed Channel Access (EDCA), which is a variant of Carrier Sense Multiple Access with Collision Avoidance (CSMA/CA) joint with Automatic Repeat Request. Specifically, before transmitting a frame, the STA senses the channel. If the channel is idle, the STA can transmit the frame. Otherwise it equiprobably draws random backoff from $\left[0,CW_{r}-1\right]$, where *r* is the retry counter (initially equal to 0), and

$$CW_{r}=\left\{\begin{array}{cc} CW_{min}, & r=0,\\ \min\left\{2CW_{r-1},CW_{max}\right\}, & r>0. \end{array}\right.$$

Here, $CW_{min}$ and $CW_{max}$ are the minimal and the maximal contention windows, respectively. By default, in 802.11ah networks, $CW_{min}=16$ and $CW_{max}=1024$.

While the channel is idle, the STA decrements the backoff every time slot σ. The backoff counter is suspended when the channel becomes busy. Once the channel becomes idle for time interval AIFS, the STA resumes counting down the backoff. When it reaches zero, the STA transmits the frame. SIFS after the arrival of the frame, the recipient STA has to send an acknowledgment (ACK). If the transmitting STA receives the ACK, the frame is considered as successfully delivered. Thus, the STA selects a new backoff value from $\left[0,CW_{min}-1\right]$ and processes the next frame, if any. If no ACK arrives within AckTimeout, the STA considers that the frame is lost. If the number of unsuccessful attempts to transmit the frame does not reach the retry limit, which is 7 by default, the STA makes another attempt. For that, it draws a new backoff value. Otherwise, i.e., if the retry limit is reached, the frame is discarded and the STA starts serving the next frame if any.

In IoT networks, it is typical that a huge number of STAs is connected to a single AP. For all of them, the start of the authentication procedure can be triggered by the same event, e.g., receipt of a beacon or a Probe Response, which results in a high contention between numerous AuthReqs. Since, at the authentication stage, the capabilities of the STAs are unknown, many control mechanisms introduced in IEEE 802.11ah—such as Restricted Access Window—are inapplicable and the STAs can rely only on the basic random channel access to transmit AuthReqs. However, the performance of random access significantly degrades with a large number of simultaneously transmitting STAs. For that reason, the IEEE 802.11ah amendment introduces two authentication control protocols: centralized and distributed ones that allow the AP to limit contention for channel access during the authentication phase.

### 2.3. Distributed Authentication Control

When the DAC protocol is used, all beacon intervals (BI), i.e., the intervals between consecutive beacons, are divided into sub-intervals called Authentication Control Slots (ACSs). To reduce contention, the STAs use a procedure which can be considered as a large-scale truncated binary exponentially backoff. Specifically, the authentication attempts are deferred by some random time which consists of *m* whole BIs and *l* whole ACSs. In other words, the STA randomly chooses a BI and an ACS within the chosen BI. At the beginning of the chosen ACS, the STA enqueues an AuthReq. To transmit AuthReq, the STAs use the default channel access, described in Section 2.2. If the STA does not succeed to authenticate, i.e., it does not receive an AuthRep within some timeout AuthenticateFailureTimeout, it increments the number of authentication attempts ρ and regenerates *m* and *l* as follows.

For each authentication attempt ρ, *m* is drawn from interval $\left[0,TI_{\rho }\right]$ and *l* is drawn from $\left[0,L\right]$, where

$$TI_{\rho }=\left\{\begin{array}{cc} TI_{min}, & \rho =0,\\ \min\left\{2TI_{\rho -1},TI_{max}\right\}, & \rho >0, \end{array}\right.$$

**Figure 2:**
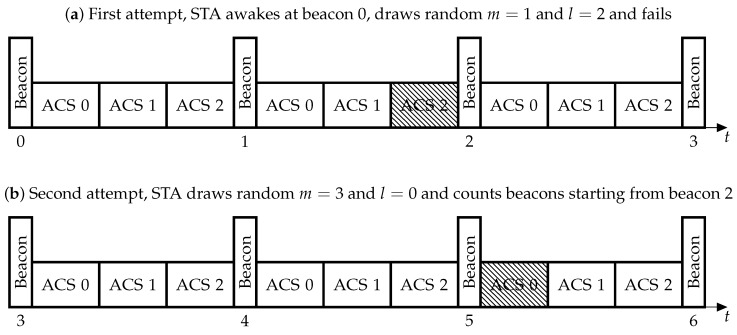
Authentication with DAC. AuthenticateFailureTimeout=1BI.

$L=\lfloor \frac{BI}{T_{AC}}\rfloor$. The ACS duration $T_{AC}$ and both the limits $TI_{min}$ and $TI_{max}$ are determined by the AP and advertised in beacons (see Figure 2).

Due to frames format, the maximal possible value of $T_{AC}$ is about 127 ms, and the maximal possible value of $TI_{min}$ and $TI_{max}$ is 255. Choosing the appropriate values for these parameters is an open question. Although keeping $TI_{max}$ at its maximal value seems reasonable, since an STA only increases its TI if the contention for channel access is too high, the impact of other parameters on performance is not apparent and is studied in the paper.

### 2.4. Centralized Authentication Control

When Centralized Authentication Control (CAC) is used, the AP dynamically changes the portion of STAs that are allowed to generate AuthReq in the current BI as follows. The beacons and probe responses carry the authentication threshold parameter: an integer number from 0 to 1023, selected by the AP. When an STA turns on, it equiprobably draws an integer value from 0 to 1022 and waits for a frame with an authentication threshold. If the drawn value is equal to or higher than the threshold, the STA waits for the next BI. Otherwise, i.e., if the drawn value is less than the threshold, the STA enqueues an AuthReq. If no AuthRep has been received within some timeout AuthenticateFailureTimeout, the STA waits for a new frame with an appropriate threshold to generate a new AuthReq.

By adaptively changing the authentication threshold sent in beacons or probe response frames, the AP can control the number of STAs trying to set up a link. In contrast to the AP, the STA generates its random value only once after being switched on and may regenerate it again only after receiving an AuthRep. It means that the drawn value is never changed during establishing a link.

The standard does not specify a way for the AP to set the authentication threshold. However, this parameter has a significant impact on the link set-up time. Specifically, to minimize the link set-up time, the authentication threshold can be set to its maximal value when the number of STAs is small. In contrast, when the number of STAs is large, the threshold should be gradually increased from its minimal to its maximal value, thus letting small portions of STAs access the channel at once. In Section 4, we design an algorithm to dynamically modify the value of the threshold.

## 3. Related Papers

Although the final version of the IEEE 802.11ah amendment [7] to the IEEE 802.11 standard was published in 2017, there are several studies of CAC and DAC performance found in the literature. Specifically, CAC was initially described in an IEEE 802.11 proposal [8], along with a simple algorithm to control authentication threshold by monitoring the length of the AP’s response queue. The algorithm is based on the following observation. If the AP and the STAs use the same contention parameters when accessing the channel and the number of STAs trying to connect to the AP is high, the rate of incoming AuthReqs may exceed the maximal possible rate with which the AP can transmit AuthReps in the channel. In other words, the AP enqueues AuthReps faster than they can be transmitted and the queue size grows. Thus, every BI, the AP increases the threshold by Δ if AP’s queue length is less than some parameter Λ; otherwise, it decreases the threshold by Δ. As shown in [6], the values of these parameters significantly affect the algorithm performance. Another drawback of this algorithm indicated in [6] is that it leads to high contention when a group of STAs suddenly appears and starts connecting to the AP. In this case, AuthReqs may be lost because of collisions and the AP will not learn for a long time because its queue is less than the given threshold.

To the best of our knowledge, paper [9] is the first published study on CAC. In this paper, the authors divide a large group of STAs into equal sub-groups, calculate the association time for a separate sub-group and then multiply the obtained time by the number of groups. Thus, they estimate the association time for the entire group and show that by selecting a certain number of sub-groups they can minimize the association time. However, the authors do not provide a way to use the authentication threshold in order to divide the STAs into groups when the AP does not know a priori the number of connecting STAs.

This problem is firstly studied in [4], where several quantities observable by the AP are considered to estimate the number of connecting STAs. The paper introduces several algorithms that allow the AP to select the authentication threshold and change it with time in order to minimize the average link set-up time for a group of STAs. The algorithms differ in the set of used quantities and in the amount of a priori available information. Simulation results show that the usage of the developed algorithms can provide an almost linear dependency of the link set-up time on the number of connecting STAs, while, without CAC, the link set-up time grows almost exponentially.

Also in [4], we have proposed an algorithm based on virtual carrier sense to provide contention-free access for the AuthRep, AReq and ARep frames.This idea has been extended and studied in detail in [10]. The authors of this paper propose to use EDCA to transmit AuthReq, and then transmit AuthRep, AReq, ARep and Ack frames separated by SIFS. To guarantee contention-free access, the STA specifies the time until the end of the Ack for ARep in the Duration/ID field of AuthReq. STAs that receive AuthReq consider the channel virtually busy for the time specified in Duration/ID field. If the AP refuses to associate the STA, the AP sets 0 in Duration/ID field of AuthRep. However, the implementation of this scheme can face multiple problems. Firstly, frames AuthReq, AuthRep, AReq, ARep must be acknowledged by an ACK and cannot themselves be used as acknowledgments. Secondly, the AP and STAs need time to process the aforementioned frames and might not be able to send them with interval SIFS. Finally, the AP can stop virtual occupancy of the channel only with a CF-end frame, in which it is impossible to send the information about a cause of association failure.

The authors of [11] develop a mathematical model of link set-up. They also present algorithms of authentication threshold selection for CAC, aimed at minimizing the average link set-up time. However, their model does not comply with the 802.11 standard because, similarly to [10], it considers a case when AuthReq and AReq are acknowledged by AuthRep and ARep, respectively, rather than by an ACK. In addition, to describe the link set-up process, the authors apply formulae for the saturated data stream with a constant successful transmission probability, while both these assumptions do not hold in case of a group of STAs connecting to an AP. The threshold control algorithms, proposed in this paper are based on the idea that the AP knows the number of STAs that should be allowed to request authentication to minimize the average link set-up time. As a result, with such an approach, hidden STAs and changes in the environment can significantly affect the efficiency of the proposed algorithms.

Apart from the provided issues, early papers [4,10,11] contain a misalignment in the CAC description with the final version of the standard that yields completely different behavior of the system of STAs and AP. Specifically, the aforementioned papers consider that an STA draws a new random value every beacon-interval, but the latest version of the 802.11ah amendment states that the STA generates a random value on network initialization and can re-draw it only after a successful authentication. Such a difference makes the proposed authentication threshold selection approaches inapplicable to connect STAs. When STAs generate random values every beacon interval, STAs have a chance to start authentication even if the AP never sets the maximal threshold value. However, in the opposite case, the AP should increase the threshold up to its maximum; otherwise, it cannot be sure that all STAs have finished link set-up.

The authors of [12] provide numerous improvements of the link set-up process. Specifically, they propose using a modification of the random access procedure for transmission of AuthReqs, based on the Sift distribution [13], and develop a closed-form analytical expression of the expected number of AuthReqs. To transmit the remaining frames (*AuthReps*, *AReq*, and *AReps*), the authors propose using a Time-Division Multiple Access (TDMA) scheme, which is an extension of our idea proposed in [4], thus ensuring the collision-free transmission. The paper also contains a modification of CAC threshold selection algorithms developed in [6]. The provided solution has been evaluated in ns-3, and altogether it yields link set-up significantly faster than the existing solution. The main drawback of this study is that the proposed improvements do not comply with the standard and require modification of both the AP and the STAs.

A step forward towards an accurate evaluation of protocols performance is the implementation of the IEEE 802.11ah Medium Access Control (MAC) and Physical Layer (PHY) in the ns-3 simulator [14] described in [15]. This implementation has been used to evaluate the performance of CAC with the algorithm proposed in [8]. Simulation results show that the link set-up time grows linearly with the number of connecting STAs. Since such an implementation of IEEE 802.11ah is being actively used to evaluate network performance in a variety of scenarios [16,17], in our research, we use this 11ah implementation, but significantly extended its functionality with new models of CAC and DAC.

In spite of numerous papers on CAC, to the best of our knowledge, only one paper [5] evaluates the performance of DAC. This paper presents a mathematical model of oversimplified DAC in a scenario when all the STAs are in the transmission range of each other. The model assumes contention-free access inside ACS, which actually violates the standard rules but simplifies calculations. Thus, an accurate performance evaluation of DAC is still an open issue, as well as the comparison of CAC and DAC in complex scenarios, which is done in the present paper.

Another (not less important) contribution of the present paper is the extension of our earlier study of CAC [6]. In that paper, we have designed a simple algorithm for CAC and showed that this algorithm provides close to optimal results in a scenario without hidden STAs when a group of STAs joins the network at once. In contrast, in the present paper, we point out the drawbacks of the previous algorithm and eliminate them, making the algorithm more robust to the varying network conditions.

## 4. Proposed Algorithm for CAC

Let us describe the proposed algorithm. Similarly to [8], we use information about the queue size and change the authentication threshold by Δ. However, the value of Δ is not constant. In contrast, it is estimated based on some learning approach.

Note that Δ determines the portion of STAs allowed authentication. For example, let *N* be the number of connecting STAs. Since the STAs equiprobably draw their integer values, when the threshold is increased by Δ, on average approximately $\frac{\Delta }{1023}N$ new STAs can start the authentication process.

The algorithm works in three modes. It starts in the *waiting* mode, in which the AP keeps the maximal threshold and monitors the queue at the end of each BI.

When the queue contains at least one AuthRep, by the end of the BI, the AP switches to the *learning* mode and sets the threshold and Δ to 1.

In the learning mode, every BI, the AP increases the threshold by Δ. To find the optimal value of Δ, we follow the classic idea to control congestion used in TCP Slow Start. Specifically, the AP doubles Δ each BI until the AuthRep queue becomes non-empty by the end of some BI, which means that we achieved congestion. At this point, the AP understands that Δ is too high and its previous value is less than or equal to the optimal one. Thus, it halves Δ and switches to the *working* mode.

In the *working* mode, each BI, the AP increases threshold by Δ if the queue is empty; otherwise, it keeps the threshold unchanged. To improve the estimation of the optimal Δ, the AP also increases Δ by 1 each BI. Such tuning is only made when the tune flag is set. This flag is reset if the queue is non-empty by the end of the BI, which indicates that the found estimation of Δ is too high. The tune flag is set when the algorithm switches from the learning mode to the working mode. Apart from that, it is also set if the queue has been empty for at least e_max consequent BIs, where e_max is the algorithm parameter.

When the threshold reaches its maximal value, the AP switches back to the *waiting* mode.

It is possible that, when the AP is in the working mode, the number of connecting STAs suddenly changes, e.g., if a new group of STAs has appeared in the network. In such a case, the estimation of the optimal Δ is not valid anymore, and the AP has to adapt to the new network conditions in order to provide fast link set-up for the STAs. The adaptation is made in the following way. In the working mode, the AP monitors the AuthRep queue length and if it becomes greater than $q_{max}$, it considers that new STAs have appeared. In such a case, the AP saves the used Δ and the current threshold value in a stack history (The stack is used since there can be several groups of STAs appearing in the network at different time.), sets Δ and the threshold to 1 and switches back to the learning mode to estimate a new optimal value of Δ, which corresponds to the number of appeared STAs. Later in the learning or in the working mode, the AP reaches the previously saved threshold value and recalculates Δ taking into account that above the old threshold the STAs from the old and the new groups have not associated yet:

$$\Delta_{new}=\frac{\Delta \times \Delta_{saved}}{\Delta +\Delta_{saved}}.$$

To explain this formula, we consider two groups of STAs of size $N_{1}$ and $N_{2}$, respectively. Let $n^{*}$ be the optimal number of STAs that should be allowed to request authentication in a BI. The optimal Δ that corresponds to that number equals $\Delta_{1}=\frac{n^{*}}{N_{1}}\times 1023$ and $\Delta_{2}=\frac{n^{*}}{N_{2}}\times 1023$, respectively. If these two groups are united, then the optimal Δ is calculated as $\Delta_{united}=\frac{n^{*}}{N_{1}+N_{2}}\times 1023=\frac{1}{1/\Delta_{1}+1/\Delta_{2}}$.

**Figure 3:**
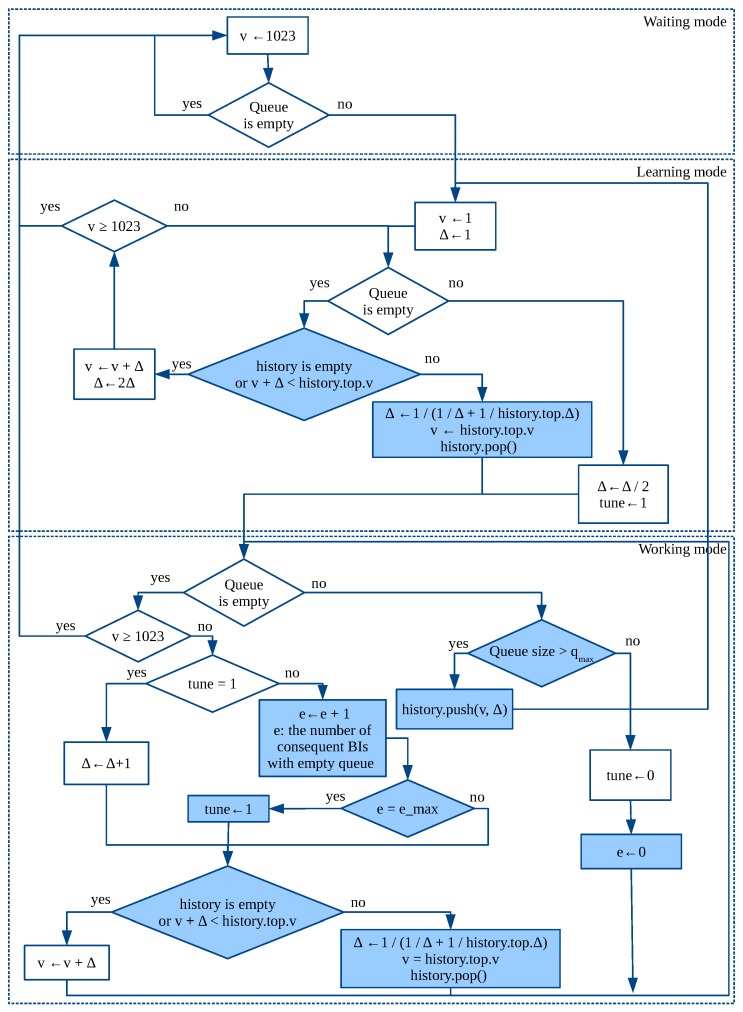
Algorithm for CAC, changes to algorithm ‘Up’ from [6] are shown with painted blocks.

The block diagram of the algorithm is shown in Figure 3, where *v* is the current threshold value and ← is the assignment operator. The block diagram uses method push that puts the provided threshold value and Δ to the top of the stack, and method pop that removes the top element from the stack.

The diagram highlights the difference with our previous algorithm, published in the paper [6].

## 5. Results and Discussion

### 5.1. Considered Scenario and Simulation Setup

To evaluate the performance of CAC and DAC, we consider a Wi-Fi HaLow network with an AP and *M* STAs (hereinafter referred to as *saturated STAs*) transmitting data in the saturated mode, i.e., they always have data frames in their queues. The saturated STAs are connected to the AP at the beginning of the experiment. In a given time instant, *N* STAs (hereinafter referred to as *new STAs*) appear and start link set-up to the AP. By default, M=20 while *N* is variable from 100 to 8000.

The AP uses one of the described authentication control protocols to limit the contention of the new STAs.

**Figure 4:**
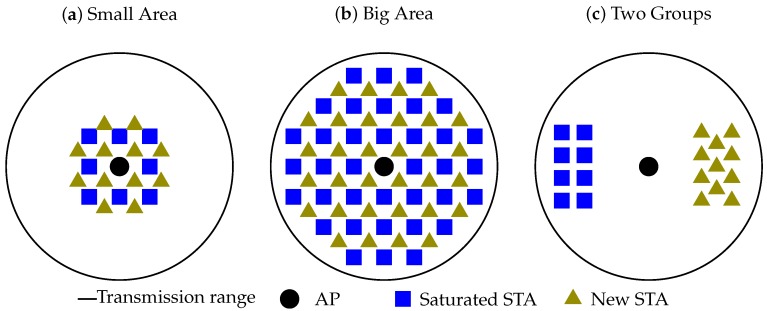
Studied cases.

We consider three cases (see Figure 4):

- Small Area: the saturated STAs and the new STAs are located close to the AP and to each other, i.e., they can clearly sense each other;
- Large Area: the saturated STAs and the new STAs are located in a wide range around the AP; thus, the central STAs sense each other, while the edge STAs cannot sense the STAs on the opposite edge;
- Two Groups: the AP can sense saturated STAs and new STAs, but saturated STAs are hidden from the new STAs.

We measure the link set-up time from the appearance of the group of new STAs until the end of association for the last STA. For that, we implement the described scenario in the ns-3 simulator [14]. The network operates in a 1 MHz channel at a fixed rate of 600 kbps. In the Small Area case, all STAs are spread uniformly within a circle with 30 m radius around the AP. In the Large Area case, all STAs are spread uniformly within a circle with 200 m radius around the AP. In the Two Groups case, saturated STAs are spread uniformly within a 20 m×20 m box at a distance of 200 m from the AP, and new STAs are spread uniformly within a 20 m×20 m box at a distance of 200 m from the AP on the diametrically opposite side. Thus, we guarantee that all the STAs can receive frames from the AP, the AP can receive frames from all the STAs, but STAs of different types do not sense each other.

At the start of the simulation, saturated STAs associate to the AP, and after successful association starts transmitting saturated data flows using EDCA. A random delay from 1 s to 5 s after all saturated STAs are associated with the AP, new STAs appear and start associating to the AP.

The AP broadcasts beacons with a period of 512 ms, and the AuthenticateFailureTimeout at STAs is set to 512 ms. It should be noted that regardless of AuthenticateFailureTimeout, once the STA starts transmission of a frame, it does not drop the frame until the frame is either transmitted or the retry limit is reached. It means that the frame generated at the beginning of a beacon-interval can still be transmitted during the next BIs, which is an important issue touched upon in Section 5.2.

### 5.2. Evaluation of Distributed Authentication Control

To evaluate the performance of DAC in the described scenario, we consider different values of its parameters, looking for the set of parameters that minimizes the link set-up time for the new STAs.

**Figure 5:**
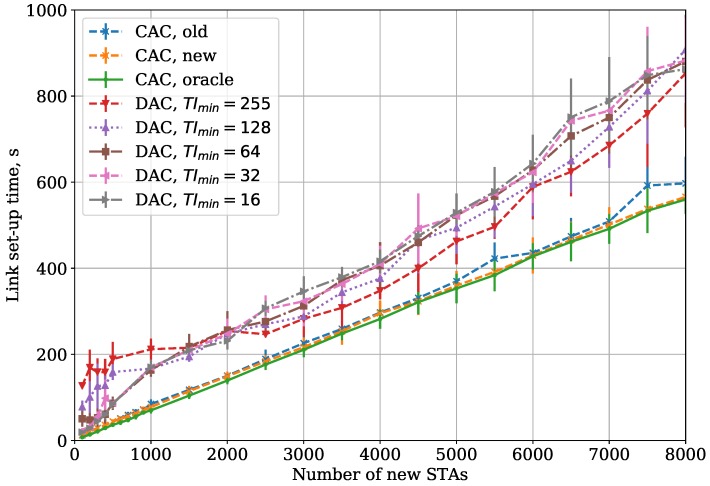
CAC and DAC protocols: dependency of the link set-up time on the number of new STAs for the Small Area case.

Firstly, we vary the $TI_{min}$ parameter with fixed $TI_{max}=255$ and $T_{AC}=60$ in the Small Area case. Figure 5 shows the dependency of link set-up time on the number of new STAs. According to the obtained results, the optimal value of $TI_{min}$ depends on the number of new STAs. When the number of new STAs is small, they can associate at the first attempt and the association lasts for $TI_{min}$ BIs on average. Thus, the use of large $TI_{min}$ is redundant and just increases the association time. At the same time, in the case of numerous new STAs, the first authentication attempts are mostly unsuccessful if $TI_{min}$ is low, which results in new authentication attempts and increases the link set-up time. Selection of high $TI_{min}$ increases the success probability of the first authentication attempt.

Curves ‘CAC’ in Figure 5 are explained in Section 5.3.

**Figure 6:**
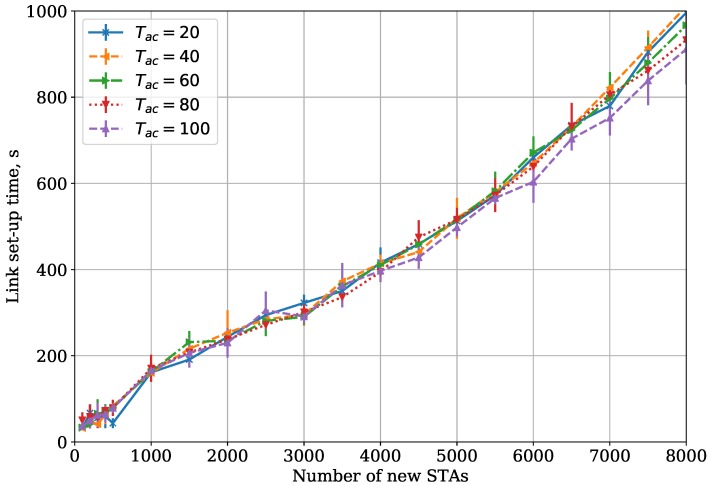
DAC protocol: dependency of link set-up time on the number of new STAs, $TI_{min}=64$ for the Small Area case.

Secondly, we vary the $T_{AC}$ parameter with fixed $TI_{min}=64$ and $TI_{max}=255$ (see Figure 6). As one can see, the variance of $T_{AC}$ has almost no effect on the link set-up time. This is caused by the fact that the STA does not necessarily transmit its AuthReq during the chosen ACS. At the beginning of its ACS, the STA just generates its AuthReq and starts the procedure of random channel access. However, if the channel is busy or in case of collisions, the STA can defer the actual transmission of AuthReq past the end of its ACS or even beyond its BI. As the result, transmission attempts are not localized within their corresponding ACSs but are spread in time regardless of the $T_{AC}$ value.

**Figure 7:**
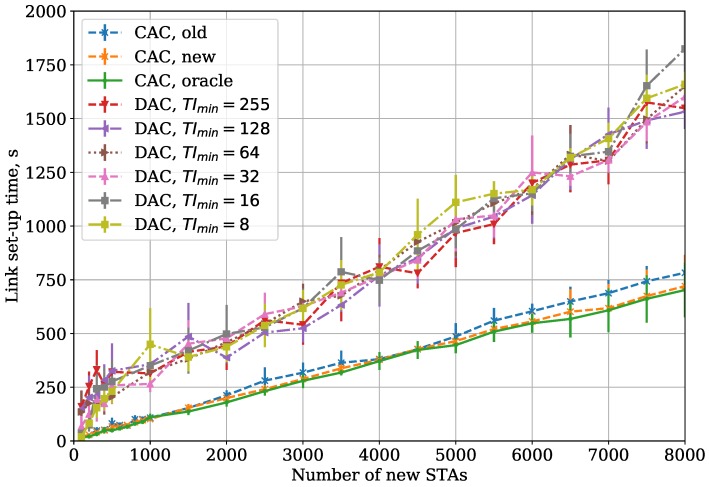
CAC and DAC protocols: dependency of link set-up time on the number of new STAs for the Large Area case.

In the Large Area case, the STAs in different parts of the network do not hear each other. It increases the frame collision rate, and, consequently, link set-up time (see Figure 7). Another effect is that the discrepancy between the curves with different parameters becomes less significant because the collisions with new STAs and with saturated STAs have a similar impact on the link set-up time.

**Figure 8:**
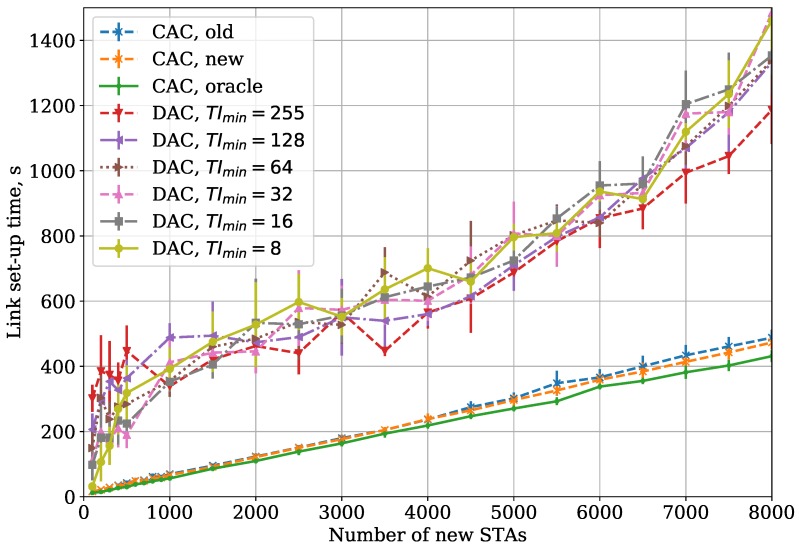
CAC and DAC protocols: dependency of link set-up time on the number of new STAs for the Two Groups case.

If the saturated STAs are hidden from the new STAs, the link set-up time becomes higher than in the Small Area case but lower than in the Large Area case (see Figure 8). The collision rate of AuthReqs and AReqs is higher than in the Small Area case, which explains increased link set-up time. At the same time, since new STAs do not sense saturated STAs, they spend less time waiting for the channel to become idle, which results in link set-up time lower than in the Large Area case. In addition, a relative increase of link set-up time is higher for small numbers of new STAs because collisions make the STAs double their TI more often.

In summary, the optimal value for $TI_{min}$ depends on the number of contending STAs, which is typically unknown at the beginning of the link set-up process. Moreover, since the impact of $TI_{min}$ on link set-up time is stronger for the small number of contending STAs, $TI_{min}$ should be rather small, e.g., 8 or 16. At the same time, the performance of the protocol almost does not depend on $T_{AC}$.

### 5.3. Comparison of Authentication Control Protocols

In this section, we compare the performance of CAC and DAC.

Figure 5 presents the dependency of the average link set-up time for a group on new STAs on the number of STAs in the Small Area case. Here, we show the link set-up time for CAC when our algorithm—described in Section 2.4—is used (curve “CAC, new”), and compare it with an old version of our algorithm, presented in [6] (curve “CAC, old”) and with Oracle, which is an idealistic solution corresponding to the case when the AP a priori knows the number of STAs that are connecting to it and sets up the authentication threshold accordingly. In other words, the results of the Oracle algorithm can be considered as a lower bound for the link set-up time. As one can see, CAC with the threshold control algorithm described in Section 2.4 is almost twice as efficient as DAC in terms of link set-up time and is very close to the Oracle solution.

In the Large Area case, we obtain a similar dependency, but the link set-up time becomes higher for all the considered protocols (see Figure 8). Moreover, the gap between the CAC and DAC link set-up time becomes more significant.

In the Two Groups case (see Figure 8), the link set-up time is higher than in the Small Area case but lower than in the Large Area case. The reason for such a difference is explained in Section 5.2.

Let us explain why CAC is much more efficient than DAC when the number of STAs is high. In case of collisions, the DAC doubles TI, therefore the time interval over which the STAs’ transmission attempts are spread is also doubled. Unlike EDCA, such deferral time is not shortened if the channel is idle. Thus, having occasionally several collisions in a row, the STA may significantly increase its link set-up time. Apart from that, the link set-up time for DAC significantly fluctuates from run to run.

Another important issue of DAC is the collision accumulation effect, which happens as follows. If $TI_{min}$ is too low, the first authentication attempts of most STAs are unsuccessful. These STAs make new attempts in a twice wider TI, but this interval intersects with the interval where the other STAs make their first transmission attempt. As a result, the collision probability for retries does not immediately decrease and TI is finally increased too much.

At the same time, when CAC is used with our authentication threshold control algorithm, the time interval and the protocol parameters are set up adaptively, in accordance with the estimated number of connecting STAs. This is why CAC allows obtaining a link set-up time close to the optimal.

**Figure 9:**
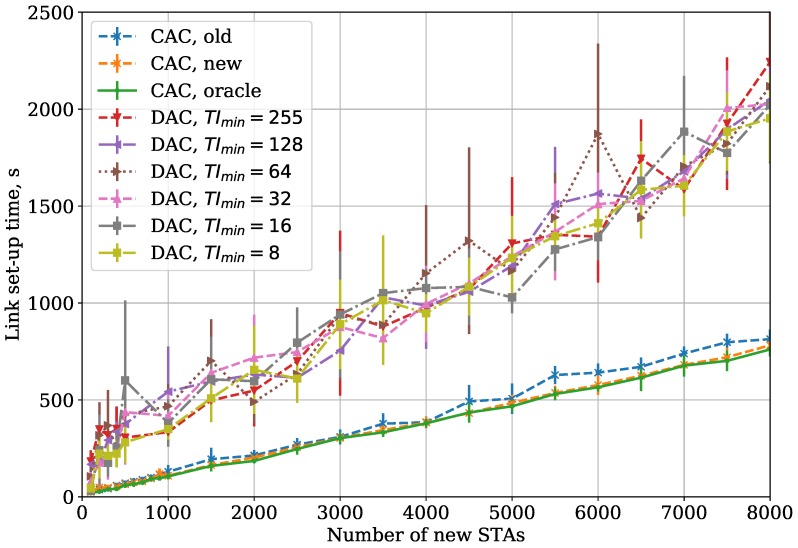
CAC and DAC protocols: dependency of the link set-up time on the number of new STAs for the Large Area case with Capture Effect.

**Figure 10:**
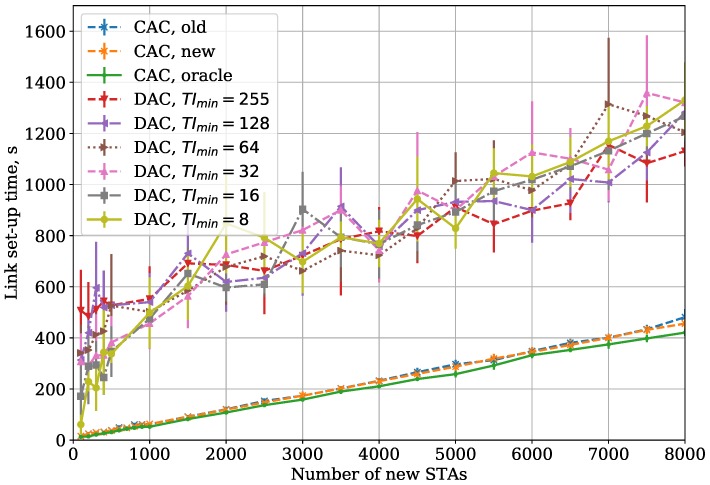
CAC and DAC protocols: dependency of the link set-up time on the number of new STAs for the Two Groups case with Capture Effect.

We also show the results for the Large Area and Two Groups cases, when the frame body capture effect is enabled. Capture effect is a phenomenon observed in some receivers [18], when an STA receiving two partially overlapping frames switches to a stronger one even if it is already receiving the weaker one. In our simulation, we considered that the switch happens if the power difference at the receiver is at least 10 dB. As shown in Figure 9 and Figure 10, with capture effect enabled, the difference between CAC and DAC increases even more. The reason for such a behavior is that, with capture effect present, the success rate for the saturated STAs rises, which in its turn makes their traffic more intensive and increases interference and collision rate for the edge new STAs. The DAC reacts to higher collision rate by increasing the average TI, which yields longer link set-up. At the same time, the CAC adapts to the collision rate and the channel occupancy and thus provides faster link set-up.

**Figure 11:**
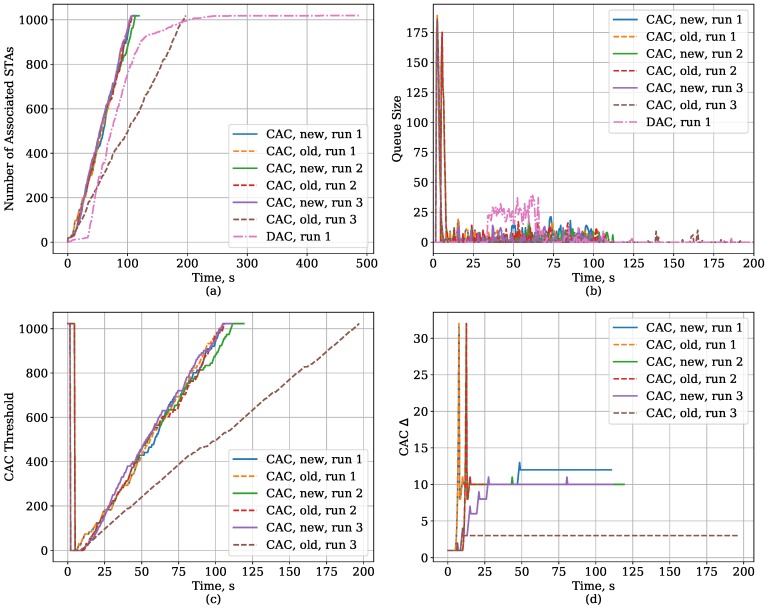
Time dependency of the CAC and DAC protocol parameters for the Large Area case with Capture Effect: (**a**) the number of associated STAs, (**b**) AP queue size, (**c**) CAC threshold, (**d**) the value of Δ in CAC.

The new version of the threshold control algorithm outperforms the old one. It provides lower link set-up time, which becomes very close to the Oracle solution, and is also more stable, i.e., it has a lower variance of the link set-up time. To explain this difference, we need to highlight again the changes we have introduced to the algorithm and consider the plots in Figure 11. They show the time dependencies of the number of STAs associated with the AP and the size of the AP queue, CAC threshold, and CAC Δ value for the old and new threshold control algorithms and for the DAC protocol. The CAC results are provided for several runs to highlight the difference between the algorithms. For the CAC protocol, the number of associated STAs grows almost linearly with time, and, for most runs, the results for the new and old algorithm are the same. When the new STAs appear, the AP queue suddenly grows, but later it is kept at a relatively low length. The CAC up algorithm quickly finds a suitable value for Δ and for the most time keeps it constant. Thus, the threshold grows linearly. However, in some runs (e.g., run 3), the old algorithm underestimates the optimal Δ, and, although lower Δ yields lower average queue size, it also yields slower growth of threshold and, as a consequence, slower link set-up time. On the contrary, the new algorithm tunes Δ if the queue has been empty for several BIs in a row. In the considered run, the algorithm firstly underestimates the optimal Δ during the learning mode, but later in the working mode increases Δ in several steps and reaches a value close to optimal.

The plots also show that the DAC protocol quickly associates most STAs, but for a small portion of STAs the link set-up time is high because they make unsuccessful authentication attempts, increase their TI and make new authentication attempts after waiting for the deferral which is the higher the more unsuccessful attempts the STA has made.

**Figure 12:**
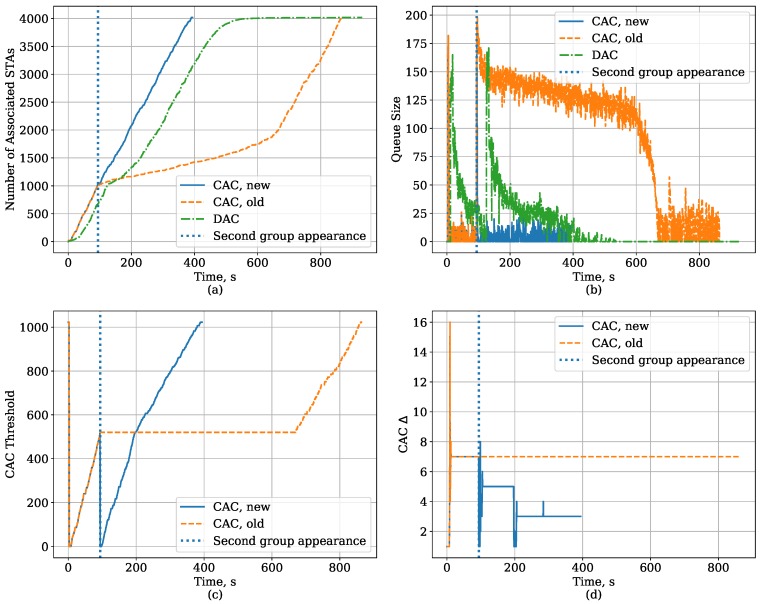
Time dependency of the CAC and DAC protocol parameters for the Large Area case with Capture Effect when new STAs arrive in two groups: (**a**) the number of associated STAs, (**b**) AP queue size, (**c**) CAC threshold, (**d**) the value of Δ in CAC.

To explain another feature of the new algorithm for the CAC protocol, we consider a situation when the new STAs arrive in two groups. Specifically, after the saturated STAs connect to the AP, a group of 2000 new STAs appears and starts associating with the AP. Later, when half of them are associated, 2000 more STAs appear and start associating too. In such a scenario, the second group of new STAs arrives while the CAC authentication threshold control algorithm is in the working mode. As shown in Figure 12, in such a case, the old version of our algorithm shows poor performance because, as soon as the second group appears, the AP experiences sudden significant increase of the queue size, freezes its threshold and waits until the queue becomes empty, which means that it waits until the STAs resolve their collisions according to bare EDCA. On the contrary, the new algorithm detects that the queue has become too long and switches back to the learning mode, drops the threshold and estimates a new Δ, thus helping the STAs to authenticate and associate without unnecessary collisions. Later, when the threshold reaches the value it had before the drop, the algorithm recalculated the Δ because the new Δ should correspond to a higher number of STAs. The effect of such a recalculation can be seen at the queue size plot, so the queue size for “CAC, new” after 200 s (when it reaches the old threshold value) is lower than the queue size for “CAC, old” after 700 s (when it unfreezes the threshold).

The changes made to the algorithm improve its ability to adapt to the number of devices in the network and decrease the link set-up time almost twofold. The new algorithm can work well even if the devices arrive in more than two groups because the algorithm maintains a history of old threshold and Δ values used in the working mode and recalculates the Δ every time it reaches them. The algorithm forgets the history only if it reaches the maximal threshold value and the queue is free, which indicates that all the STAs have set up the link with the AP.

It should be noted that, in this scenario, for most STAs, the DAC provides a lower link set-up time than the old CAC authentication threshold algorithm because the new STAs randomize the BIs when they start their authentication attempts. However, the DAC still suffers from the fact that a small number of STAs have very high link set-up time. In addition, it is less efficient than our new algorithm.

To sum up, since DAC does not require any additional algorithms, it is much easier in implementation. However, its ability to adapt to the current situation in the network is rather limited. With the standard default parameters ($TI_{min}=8$, $TI_{max}=255$, $T_{ac}=10$), it provides the link set-up time up to four times higher than the theoretically lower bound.

At the same time, with our authentication threshold control algorithm, the link set-up time for CAC grows almost linearly and is rather close to the theoretical lower bound. The designed algorithm is robust to the changing number of associating STAs.

## 6. Conclusions

We have studied the link set-up process in Wi-Fi HaLow networks, which consists of two main handshakes: Authentication and Association. Both handshakes are performed using Wi-Fi random channel access, the performance of which significantly degrades in case of a high number of contending STAs. Such a situation is typical for Wi-Fi HaLow networks because this technology has been designed as a version of Wi-Fi for the Internet of Things scenarios, so the Wi-Fi HaLow has two possible solutions to limit the contention for channel access, namely, CAC and DAC.

With CAC, the AP periodically broadcasts the Authentication Threshold, the increment of which effectively determines the percentage of devices that are allowed to start authentication at the moment. In this paper, we have proposed a new algorithm for CAC, which is both efficient and robust. We tested this algorithm for the case when, besides connecting STAs, there are STAs which transmit saturated data flows. When they are hidden from the connecting ones, the algorithm preserves its efficiency even in such an unfriendly scenario. This is the first major contribution of this paper.

The second contribution is related to DAC. With DAC, the STAs randomly select intervals, during which they start authentication and, in case of failure, make new attempts after waiting a number of intervals, chosen according to the binary-exponential approach, while the AP broadcasts parameters of these intervals. We have run the excessive simulation to determine the best configuration of DAC and have shown that there is no set of parameters that can minimize the link set-up time for all possible numbers of connecting STAs, and that, in some scenarios, the DAC is essentially insensitive to some of its parameters, which is valuable for optimal configuration.

The third contribution of this paper is the comparison of the performance of CAC with DAC in different complex scenarios. We have shown that CAC outperforms DAC. However, such an advantage of CAC comes at the cost of complexity of the protocol and the need for the AP to constantly track the link set-up process, which can be done with the designed algorithm.

As a direction of future work, we consider the performance evaluation of CAC and DAC in a case with multiple APs, where the inter-network interference might affect the link set-up time of the devices, and such problems as load balancing among the APs, minimization of service traffic and handover optimization should be solved to provide the fast association of devices and to improve the overall network performance.

## Abbreviations

The following abbreviations are used in this manuscript:

ACK	Acknowledgement Frame
ACS	Authentication Control Slot
AIFS	Arbitration Inter-Frame Space
AP	Access Point
AID	Association ID
BI	Beacon Interval
CAC	Centralized Authentication Control
DAC	Distributed Authentication Control
EDCA	Enhanced Distributed Channel Access
IoT	Internet of Things
MAC	Medium Access Control layer
PHY	Physical layer
RAW	Restricted Access Window
SIFS	Short Inter-Frame Space
STA	Station
